# Multi-Omic Investigation of Low-Nitrogen Conditional Resistance to Clubroot Reveals *Brassica napus* Genes Involved in Nitrate Assimilation

**DOI:** 10.3389/fpls.2022.790563

**Published:** 2022-02-11

**Authors:** Yoann Aigu, Stéphanie Daval, Kévin Gazengel, Nathalie Marnet, Christine Lariagon, Anne Laperche, Fabrice Legeai, Maria J. Manzanares-Dauleux, Antoine Gravot

**Affiliations:** ^1^IGEPP, INRAE, Institut Agro, Université de Rennes 1, Le Rheu, France; ^2^P2M2 PRP, BIA, INRAE, Le Rheu, France

**Keywords:** oilseed rape, Plasmodiophora, transcriptome, fertilization, disease resistance, NRT2, salicylic acid

## Abstract

Nitrogen fertilization has been reported to influence the development of clubroot, a root disease of *Brassicaceae* species, caused by the obligate protist *Plasmodiophora brassicae*. Our previous works highlighted that low-nitrogen fertilization induced a strong reduction of clubroot symptoms in some oilseed rape genotypes. To further understand the underlying mechanisms, the response to *P. brassicae* infection was investigated in two genotypes “Yudal” and HD018 harboring sharply contrasted nitrogen-driven modulation of resistance toward *P. brassicae*. Targeted hormone and metabolic profiling, as well as RNA-seq analysis, were performed in inoculated and non-inoculated roots at 14 and 27 days post-inoculation, under high and low-nitrogen conditions. Clubroot infection triggered a large increase of SA concentration and an induction of the SA gene markers expression whatever the genotype and nitrogen conditions. Overall, metabolic profiles suggested that N-driven induction of resistance was independent of SA signaling, soluble carbohydrate and amino acid concentrations. Low-nitrogen-driven resistance in “Yudal” was associated with the transcriptional regulation of a small set of genes, among which the induction of *NRT2*- and *NR*-encoding genes. Altogether, our results indicate a possible role of nitrate transporters and auxin signaling in the crosstalk between plant nutrition and partial resistance to pathogens.

## Introduction

Nitrogen fertilization has been studied for a long time in relation with its impact on plant diseases (reviewed in Huber and Watson, 1974; Walters and Bingham, 2007; Dordas, 2008; Fagard et al., 2014; Mur et al., 2017; Sun et al., 2020). Although it has been early recognized that the chemical form of nitrogen fertilizer matters, there are also important quantitative concerns. It was indeed often expected—*a priori*—that excessive nitrogen levels tend to promote the severity of plant diseases (Chen et al., 2007; Devadas et al., 2014; Huang et al., 2017). However, the reverse has been also reported, then no general pattern applies to all pathosystems (Brennan, 1993; Krupinsky et al., 2007; Lecompte et al., 2010; Fagard et al., 2014; Précigout et al., 2017). Among the reasons most frequently given, the levels of organic or inorganic nitrogen in plant tissues can directly affect the development of pathogen for basic nutritional reasons (Talbot et al., 1997; Snoeijers et al., 2000). Fertilization can also indirectly influence plant-signaling pathways, promoting or depreciating plant ability to trigger relevant defense responses (Ding et al., 2021). Finally, plant nitrogen status exerts sometimes a critical influence on the cellular physiology of pathogen itself, modulating part of virulence functions (Pérez-García et al., 2001; Donofrio et al., 2006; Bolton and Thomma, 2008; López-Berges et al., 2010; Huang et al., 2017; Soulie et al., 2020; Gazengel et al., 2021). The varying nitrogen supply can damper or enhance the resistance level for specific genotypes and for specific nitrogen conditional resistance loci (Ballini et al., 2013; Thalineau et al., 2017). However, for many diseases, such as clubroot, the nitrogen effect is not well characterized.

Clubroot is a worldwide root disease affecting all *Brassicaceae* species, caused by the obligate biotrophic parasite *Plasmodiophora brassicae*. The life cycle of *P. brassicae* starts with a primary infection phase in the root hairs, followed by a secondary infection inside cortical and stele cells, triggering cell proliferation, and leading to the development of root galls (Tommerup and Ingram, 1971; Kageyama and Asano, 2009). Genetic resistance plays a pivotal role in the management of clubroot disease in *Brassica* crop productions (reviewed in Piao et al., 2009; Hatakeyama et al., 2017; Dakouri et al., 2021). However, efforts are faced to a steady rise of resistance breaking, and a better understanding of clubroot resistance (including partial quantitative resistance) is needed to support knowledge-based breeding and management of resistant varieties. In parallel, increasing omics-based efforts have been carried out to address the molecular and biochemical basis of clubroot responses. Those works have highlighted the importance of a series of regulatory processes associated with plant primary and secondary metabolism (Pedras et al., 2008; Siemens et al., 2011; Wagner et al., 2012), the role of jasmonic acid (JA) and salicylic acid (SA)-driven defense responses in the inhibition of symptom development (Agarwal et al., 2011; Jubault et al., 2013; Lemarié et al., 2015) and the central role of auxin, cytokinin, and brassinosteroid-driven regulations (Devos et al., 2006; Siemens et al., 2006; Agarwal et al., 2011; Schuller et al., 2014).

We recently identified (Laperche et al., 2017; Aigu et al., 2018) a series of *B. napus* genotypes, of which the genotype “Yudal,” displaying partial resistance to clubroot when cultivated under low nitrate supply, but for which partial resistance was almost lost when switching to high nitrate input. Low-nitrogen-triggered resistance was not systematically observed in the progeny of “Yudal,” and a linkage genetic analysis identified a couple of resistance QTL for which the relative effect of resistance alleles from “Yudal” was conditional to nitrogen fertilization level (Laperche et al., 2017; Aigu et al., 2018). However, cellular and molecular elements underlying such genotype x environment crosstalk remain yet largely unknown.

The aim of the present study was to understand the plant molecular processes associated with the modulation of clubroot response under low-nitrogen conditions in the *B. napus* genotype “Yudal.” Nitrogen-dependent cellular responses to infection were analyzed in “Yudal” and in the genotype HD018 used as control. HD018 was chosen among a double haploid progeny from the cross between “Darmor-*bzh*” and “Yudal” (Foisset et al., 1996; Laperche et al., 2017) among the genotype subset displaying stable high susceptibility level whatever the amount of nitrate supplied (Laperche et al., 2017). Transcriptional and metabolic responses, particularly those associated with primary and defense metabolism, were investigated in inoculated and non-inoculated roots of both “Yudal” and HD018 at 14 and 27 days post-inoculation (dpi). Results were analyzed with the double prospect of: 1/identifying genotype responses to low-nitrogen supply and *P. brassicae* inoculation, by focusing on transcriptional and biochemical responses associated with primary metabolism and plant defense and 2/identifying contrasted genotype-dependent responses to the specific combination of abiotic (nitrogen) and biotic (*P. brassicae* infection) constraints potentially explaining the low-nitrogen conditional resistance.

## Materials and Methods

### *Plasmodiophora brassicae* and Plant Material

The *P. brassicae* selection isolate eH (Fähling et al., 2003), belonging to pathotype 1, as defined by (Somé et al., 1996), was propagated on Chinese cabbage (*B. rapa* spp. *pekinensis* cv. Granaat). *B. napus* cv. “Yudal,” a spring oilseed rape, and HD018, a doubled haploid (DH) from the “Darmor-*bzh*” x “Yudal” progeny (Foisset et al., 1996), were used for the RNA-seq and metabolic analyses.

### Clubroot Assay Under Low-(N1) and High (N8)-Nitrogen Condition

Seeds of “Yudal” and HD018 were sown in 4-cm-diameter pots (1 seed per pot) filled with “Falienor 922016F3” potting medium (Falienor, Vivy, France), which consists of 65% Irish peat, 20% black peat, 15% perlite, and 2% clay. The pots were transferred to a phytotron maintained at temperatures ranging from 19 to 22°C under a 16/8 h day/night cycle. Two Hoagland-based nutrient solutions containing 1 mM (thereafter called N1 condition) or 8 mM (N8 condition) of nitrate, were supplied to the plants by watering at the bottom of the pots (sub-irrigation) according to Laperche et al. (2017). Plants were sub-irrigated with the appropriate nutrient solution twice a week from sowing to 21 days after sowing and then three times per week until the end of the assays. Inoculations were performed 1 week after sowing by applying 1 ml of resting spores solution (1 × 10^7^ spores/ml) as previously described by Manzanares-Dauleux et al. (2000). Experiments were conducted in four replicates. Macroscopic root symptoms were observed, and a disease index was determined at 27 and 42 days post-inoculation (dpi) according to the method of Buczacki et al. (1975) as modified by Manzanares-Dauleux et al. (2003). Root samples were collected 14, 27, and 42 dpi. Root samples were from 24 plants at 14 dpi, 12 plants at 27 dpi, and 6 plants at 42 dpi, for each combination of nitrogen level, genotype, inoculation and for each of the four replicates (total = 32 samples per time-course point). Samples were quickly frozen in liquid nitrogen, then freeze-dried, and grounded with a FastPrep-24 (MP biomedicals).

### Metabolic Profiling

#### Non-structural Carbohydrates, Polyols, Organic Acids, and Amino Acids Quantification

For non-structural carbohydrates, polyols, organic acids, and amino acids profiling, 10 mg of the freeze-dried root powder per sample was used. A methanol–chloroform–water-based extraction was performed according to the following procedure: ground samples were suspended in 500 μl of methanol containing two internal standards: 200 μM 3-aminobutyric acid (BABA; for amino acid quantification) and 400 μM adonitol (GC analysis). Suspensions were agitated for 15 min at room temperature. Then, 250 μl of chloroform was added, followed by a 10 min agitation step. Finally, 500 μl of water were added, and samples were vortexed and centrifuged at 12,000 g for 5 min to induce phase separation. The upper phase, which contained non-structural carbohydrates, polyols, organic acids and amino acids, was transferred to a clean microtube and used for subsequent analysis.

For amino acid profiling, 50 μl of each methanol–water extract was dried under vacuum. Dry residues were suspended in 50 μl of ultrapure water, and 10 μl was used for the derivatization employing the AccQ-Tag Ultra derivatization kit (Waters, Milford, MA, United States). Derivatized amino acids were analyzed using an Acquity UPLC-DAD system (Waters) according to Albert et al. (2012). BABA was used as internal standard.

For non-structural carbohydrates, polyols, and organic acids profiling, 50 μl of polar extract were dried under vacuum. The dry residue was dissolved in 50 μl of 20 mg ml^−1^ methoxyamine hydrochloride in pyridine at 30°C for 90 min under orbital shaking. Then, 50 μl of N,O-bis(trimethylsilyl)trifluoroacetamide (BSTFA) were added and samples were incubated at 37°C for 30 min and then at room temperature overnight before injection in GC-flame ionization detection (FID) according to Lugan et al. (2009). Ribitol was used as internal standard.

#### Salicylic Acid and Jasmonic Acid Quantification

SA and JA were quantified starting from 20 mg of the freeze-dried root powder per sample. After addition of 1 ml of a methanol: water: formic acid (80:19:1 by vol.) mixture solvent, tubes were ultrasonicated and agitated at room temperature for 30 min. The tubes were then centrifuged at 12,000 *g* for 10 min, and the supernatants were removed into new 1.5 ml tubes. The pellets were re-extracted with 1 ml of the extraction solvent, and the supernatants were pooled and dried in a speed vacuum centrifuge. Dried residues were then resuspended in 100 μl of acidified methanol. Phytohormones were analyzed by injecting 5 μl of resuspended samples in an Acquity UPLC system (Waters) coupled to a triple quadrupole detector equipped with an electrospray ionization (ESI) source according to Fan et al. (2011). Authentic SA and JA (Sigma-Aldrich and Olchemim Ltd.) were used as external standards.

### RNA-Seq Analyses

#### RNA Isolation, cDNA Library Construction, and Sequencing

RNA was extracted from 20 mg of freeze-dried roots powder using TRIzol reagent (Invitrogen, Carlsbad, CA, United States) according to the manufacturer’s instruction. RNA purity was determined using a 2,100 bioanalyzer (Agilent, Santa Clara, CA, United States). cDNA library preparation and sequencing were conducted by GenoScreen in Lille, France. mRNA was isolated and fragmented. These short fragments of mRNA were used as templates to synthesize cDNA with random primer. cDNA end was repaired to permit Illumina sequencing adaptor. DNA libraries were generated by PCR amplification and controlled by fragment analyzer kit “DNF 474 High Sensitivity NGS Fragment.” A total of 64 libraries were created, based on all the samples from 14- and 27-dpi. These libraries were paired-end sequenced (2×150 bp) using an Illumina HiSeq™ 4,000.

#### Read Mapping and Counting

High-quality paired reads were mapped against the *B. napus* genome cv. “Darmor*-bzh*” (Version 4.1; Chalhoub et al., 2014), using STAR, version 2.5.2a (Dobin et al., 2013). The mapping parameters were adjusted according to the properties of the polyploid *B. napus* genome, maximum 6 multimapping, and the divergence between genotypes used for RNA-seq and reference genome, maximum 6 mismatch for 300 pb. The reads aligned to *B. napus* genome were used to generate gene read counts based on *B. napus* annotation (Version 5) with FeatureCounts, version 1.5.0 (Liao et al., 2014). For the counting, read equally best mapped to more than one location in the reference genome, but below seven, were count fractionally.

#### Differential Expression

The R package EdgeR (Robinson et al., 2010) was used to identify differential gene expression. The raw read counts, calculated with FeatureCount, were used as the input of EdgeR. Genes with expression level below 0 log^2^ CPM (Count Per Million) were removed. The signal for each gene was normalized using TMM (Trimmed Mean of M values) and CPM normalization. Differential expression analyses were performed with EdgeR exact test, according to the evidence of Schurch et al. (2016).

#### Gene Ontology Term Enrichment

GO term enrichment analysis was performed with agriGO v2.0 (Tian et al., 2017), using Fisher’s exact statistic test and Yekutieli multi-test adjustment method (value of *p* < 0.05). GO term analysis was based on *Arabidopsis thaliana* database, using gene homology with *B. napus*.

## Results

### Gall Development Was Inhibited Since 27 dpi Under Low-Nitrogen in “Yudal”

**Figure 1:**
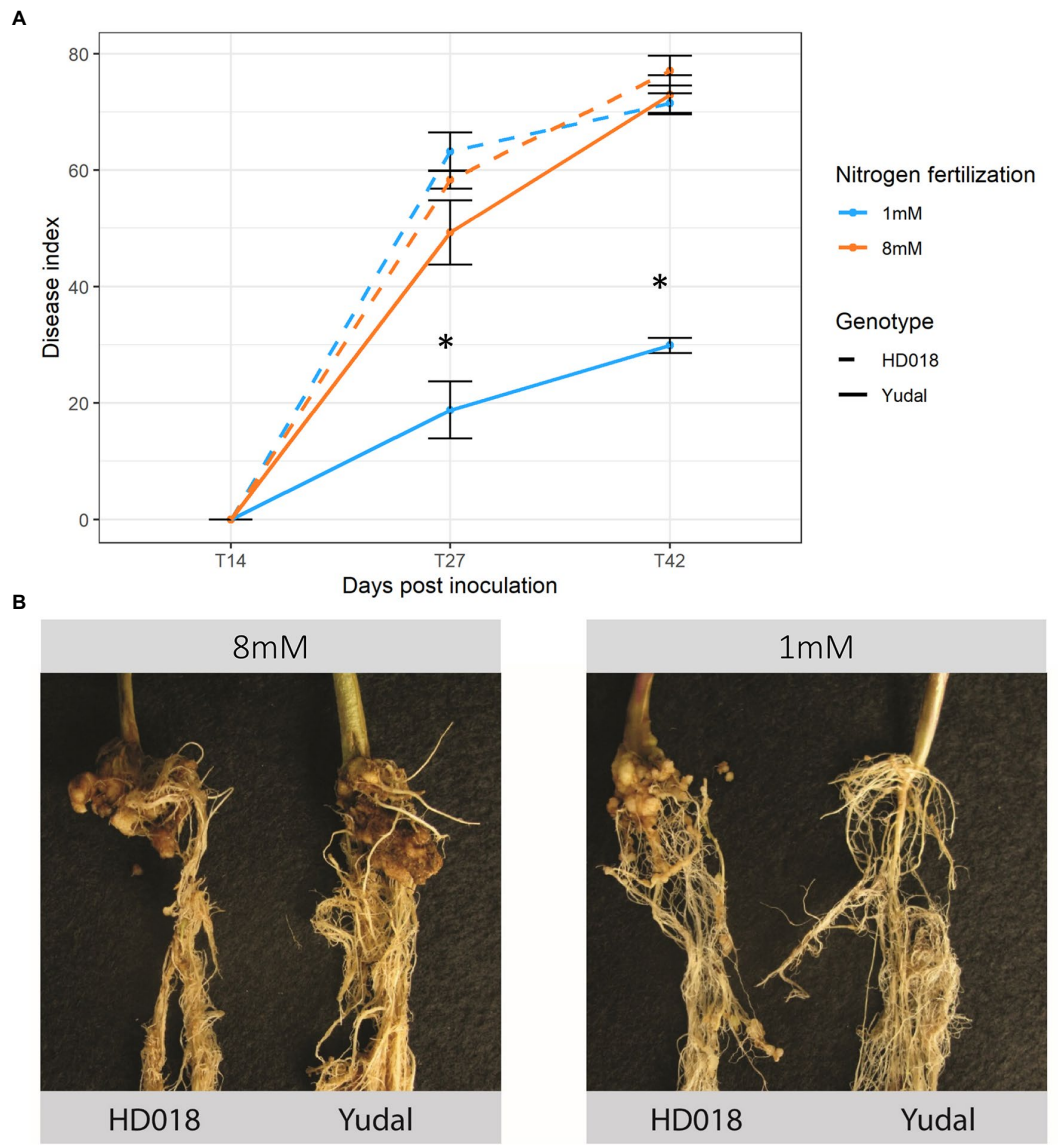
Root symptoms development induced by inoculation with eH isolate of *Plasmodiophora brassicae* in the two *Brassica napus* genotypes, “Yudal” and HD018, under two nitrogen supplies, 1 and 8 mM. **(A)** Disease index at 14, 27, and 42 days post-inoculation (dpi). Standard error are represented. *Represent statistical significant difference according to Duncan’s multiple range test, *p* < 0.05 **(B)** Root symptoms at 49 dpi.

Clubroot symptoms were not apparent at the first time-course point 14 dpi, then increased between 27 and 42 dpi (Figure 1A). Under high-nitrogen supply (N8 condition), “Yudal” and HD018 displayed similar levels and kinetics of clubroot symptom development, reaching DI = 73 and DI = 77 at 42 dpi, respectively. Under low-nitrogen supply (N1 condition), significantly fewer macroscopic symptoms on “Yudal” roots (DI = 19 at 27 dpi, then DI = 30 at 42 dpi), were observed relatively to N8 condition. By contrast, in the HD018 genotype clubroot symptoms were identical whatever fertilization treatment. Forty-nine dpi, very small galls were observed in “Yudal” roots under N1, while in the other 3 conditions (“Yudal” under N8, and HD018 under N1 and N8), large galls developed on the roots (Figure 1B).

### Plant Genotype Is the Major Driver of Transcriptome Differences Between Samples, Followed by Inoculation, Then to a Minor Extent by Nitrogen

The transcriptional regulations patterns of “Yudal” and HD018 were analyzed by RNA-seq at 14 and 27 dpi according to inoculation factor (non-inoculation and inoculation) and fertilization factor (N1 and N8; Supplementary Table S1). A total of 3,025,412,897 reads generated by 150-bp paired-end sequencing from the 64 samples (average of 47,272,076 reads per sample) were conserved after the quality control process (Q > 30% and read length > 50 bp). Approximately 83% of these reads (2.5 billion mapped reads) were aligned on the genome of *B. napus*. Similar distribution of the *B. napus* genes expression level was observed for the 64 samples, suggesting that no bias was introduced during the construction of cDNA libraries (Supplementary Figure S1). A total of 56,754 genes of *B. napus* were considered as expressed (Log2CPM > 0 in at least 4 samples). For information purposes, using separately HD018 and “Yudal” samples, a total of 55,198 and 53,933 genes were, respectively, considered as expressed, including 52,910 genes common to both genotypes.

The similarities and differences between RNA-seq samples were represented using MultiDimensional Scaling plot (Supplementary Figure S2). In our dataset, samples were clearly clustered according to the genotype along the first dimension of MDS (49% of total variance) and according to the inoculation factor (inoculated vs. non-inoculated) along the second one (11% of total variance’ Supplementary Figure S2A). Sample clustering along the third MDS axis was reflecting combinations of nitrate fertilization and time-course points (7% of total variance’ Supplementary Figure S2B). Summary of Differentially Expressed Genes (DEGs) between genotypes, inoculation, and nitrogen factors was indicated in Supplementary Table S2, for 14 and 27 dpi.

### Pathogen Inoculation Induced Similar SA-Related Defense Response Patterns in Both Genotypes, Whatever Nitrogen Supply

**Figure 2:**
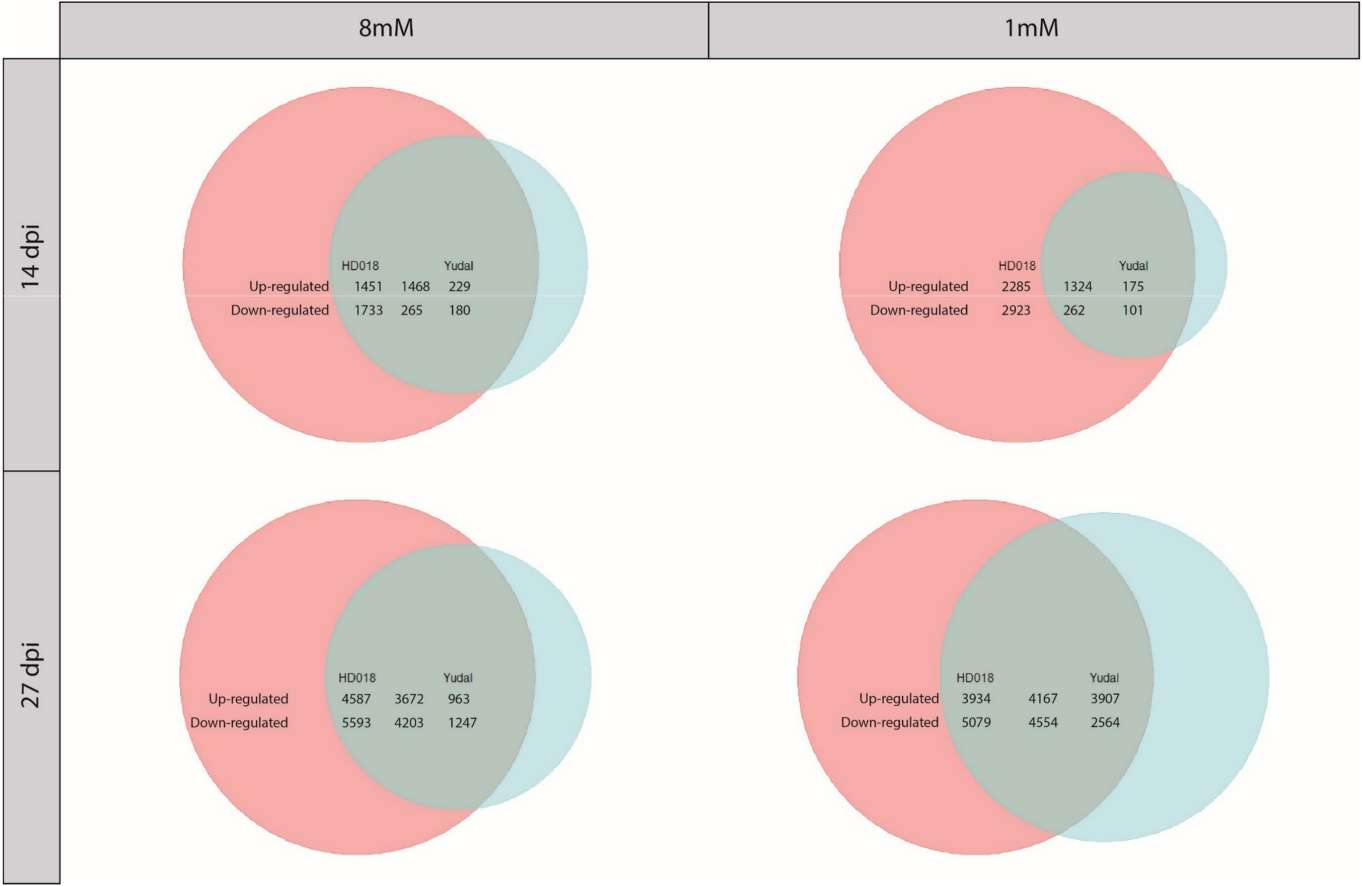
Venn diagrams of differentially expressed genes under inoculation condition compared to non-inoculated condition in the two *Brassica napus* genotypes, “Yudal” and HD018, under two nitrogen supplies, 1 and 8 mM. Pink and blue circles represented genes differentially expressed in HD018 and “Yudal,” respectively. Green part obtained by the superposition of pink and blue circles represents the differentially expressed genes common to both genotypes. In green part, only the genes with same type of regulation are considering. For each Venn diagram, the genotype with the higher amount of DEGs is represented by a circle of the same size (maximum size, independently of the number of gene). Then, the sizes of the two other color (other genotype and common part) are proportional to the biggest circle based on their amounts of genes included into. The numbers show exactly how many genes are upregulated or downregulated for each of the three colored parts.

Under N8, clubroot infection in HD018 resulted in the transcriptional regulation of 4,917 genes at 14 dpi and 18,055 genes at 27 dpi (Figure 2). Under the same nitrogen supply, 2,142 genes at 14 dpi and 10,085 genes at 27 dpi were transcriptionally regulated by clubroot infection in “Yudal.” An important proportion of these DEGs was common to both genotypes (39% of DEGs by infection were induced or repressed in both genotypes at 14 and 27 dpi). More importantly, the “Yudal” DEGs set was largely included in HD018 DEGs set (81% at 14 dpi and 78% at 27 dpi).

Under N1, clubroot infection in HD018 resulted in the transcriptional regulation of 6,794 genes at 14 dpi and 17,734 genes at 27 dpi (Figure 2). Under the same condition, 1,862 genes at 14 dpi and 15,192 genes at 27 dpi were transcriptionally regulated by clubroot infection in “Yudal.” An important proportion of these DEGs was common to both genotypes (22 and 36% of DEGs by infection were induced or repressed in both genotypes at 14 and 27 dpi, respectively), and as in N8 condition, the “Yudal” DEGs set was largely included in HD018 DEGs set (85% at 14 dpi and 57% at 27 dpi).

A GO term-based analysis with AgriGo revealed that the sets of clubroot-triggered and clubroot-repressed genes in both genotypes were enriched in similar biological process, independently of time-course point and nitrogen fertilization. The set of clubroot-triggered genes was enriched in biological processes including protein amino acid phosphorylation, defense response to fungus and bacterium, systemic acquired resistance (SAR), and response to SA stimulus. The set of clubroot-repressed genes was enriched in biological process that included glucosinolate biosynthetic process and lipid localization. A detailed list of the genes associated with those GO terms and that were differentially expressed in our dataset are given in the Supplementary Table S3A.

**Figure 3:**
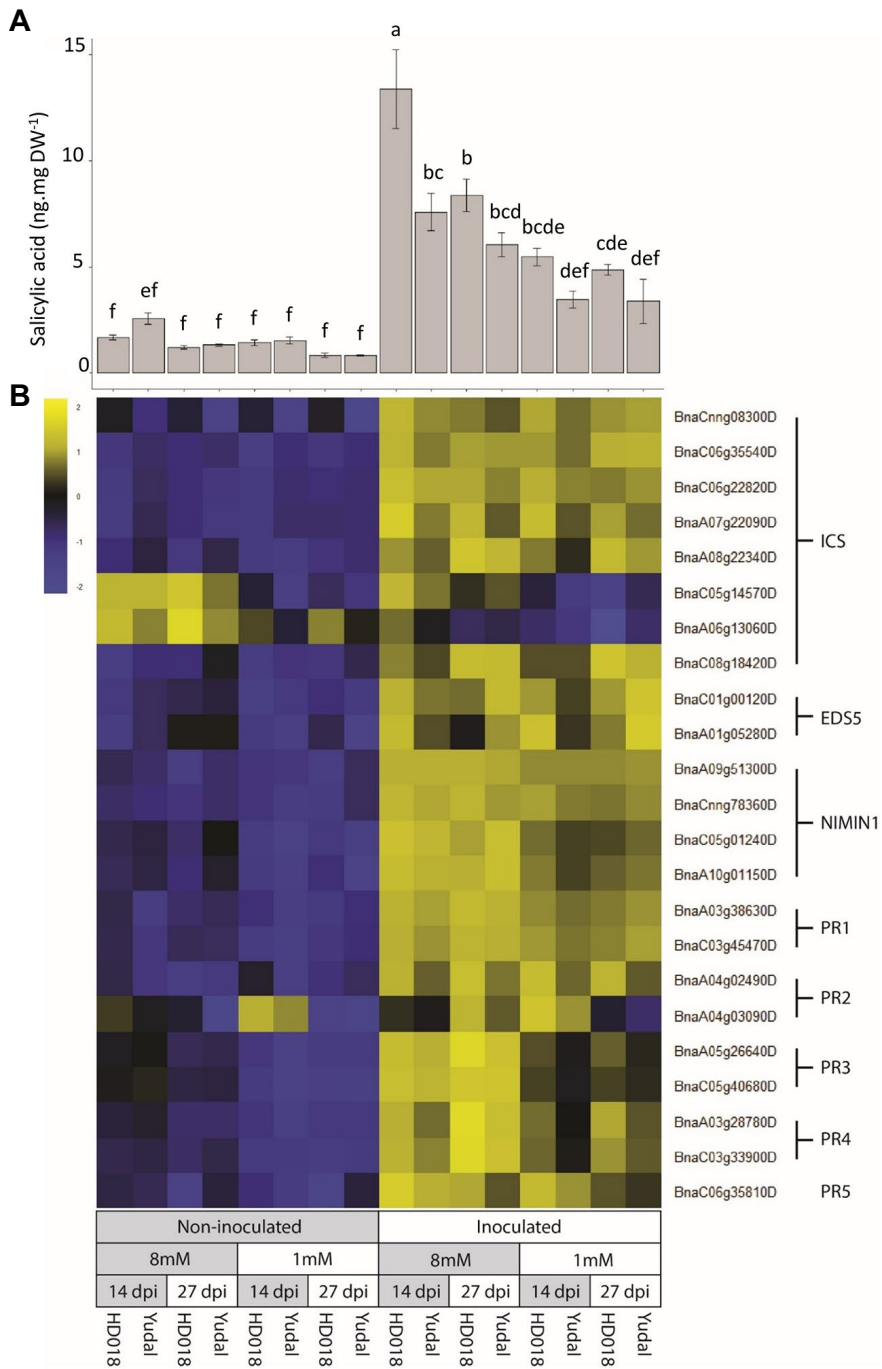
Salicylic acid (SA) response induced by *P. brassicae* inoculation. **(A)** SA content in root samples. Standard error are represented. Values followed by the same letter (a, b, c, d, e, f ) are not significantly different (*p*<0.05) by Duncan’s multiple range test. **(B)** Expression of genes related to SA biosynthesis and SA resistance. The gene expression data were centered and reduced. ICS: Isochorismate synthase, EDS5: Enhanced disease susceptibility 5, NIMIN1: Non-inducible immunity interacting 1, PR: Pathogenesis-related gene.

To get a further validation about the importance of SA-related responses during plant responses to clubroot infection, SA was quantified and the expression of a selection of classical SA-related marker genes was studied more specifically. SA concentration was increased in the inoculated roots in response to infection by *P. brassicae* compared to non-inoculated roots (Figure 3A). This accumulation was observed in both genotypes, and furthermore, no significant difference was observed between inoculated “Yudal” and HD018 under N1 condition at the two time-course points. The expression of SA-related marker genes was also induced by inoculation in all the samples (Figure 3B). Four copies of *NIMIN1*, a NPR1-interacting protein involved in immune responses, were induced by pathogen infection (with a mean log2FC of 3.5). Six *B. napus* orthologues of the *A. thaliana ISOCHORISMATE SYNTHASE* (*ICS*), coding a key enzyme of SA biosynthesis, were induced by pathogen infection (with a log2FC of 0.82). Two orthologous of the *ENHANCED DISEASE SUSCEPTIBILITY 5* (*EDS5*) gene of *A. thaliana*, implicated in SA transport from chloroplast to cytoplasm, were significantly upregulated in inoculated roots (with a mean log2FC of 1.4). Accordingly, expression of typical SA-responsive gene markers was significantly higher in the inoculated root samples under high-nitrogen, compared to non-inoculated root samples. Pathogen-related (PR) genes, implicated in SA response, particularly two gene copies of PR1 and one ofPR5 were induced by the infection under N8 condition with a mean Log2FC of 10.4 and 5.54, respectively. The same analysis was carried out on JA-related response. However, *P. brassicae* inoculation was not inducing any JA-related response in any genotype (Supplementary Figure S3).

SA response level was not higher in “Yudal” under low-nitrogen condition compared to the other inoculated samples. Thus, hormone profiling and transcriptomic data gave no support to the hypothesis that resistance to clubroot of “Yudal” under N1 could be related to the extent of SA responses.

### Variations of Nitrate Supply Did Not Trigger Defense and Induced Similar Responses in Non-inoculated “Yudal” and HD018

**Figure 4:**
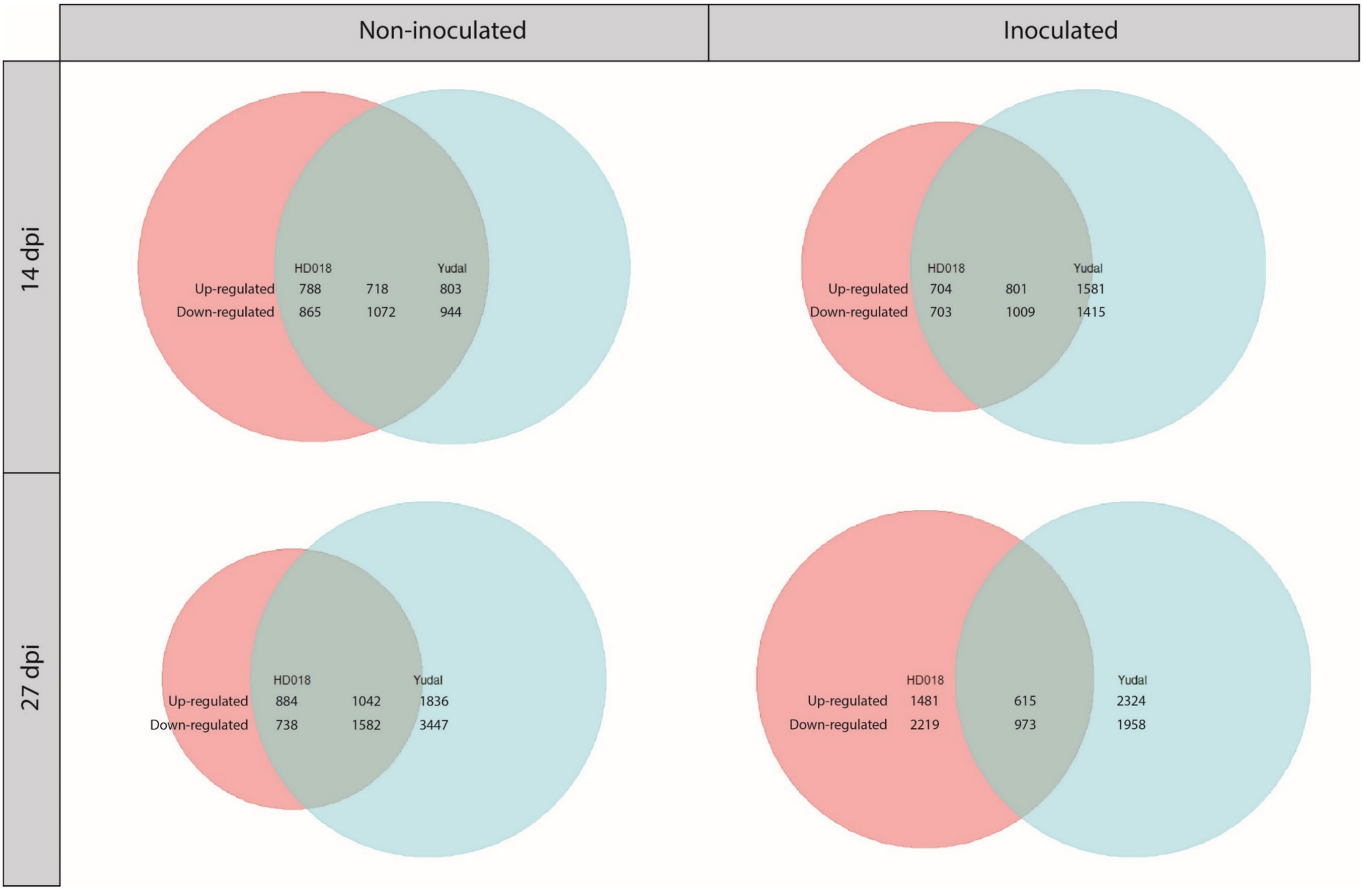
Venn diagrams of differentially expressed genes under 1 mM condition compared to 8 mM condition in the two *Brassica napus* genotypes, “Yudal” and HD018, under two inoculation condition, non-inoculated and inoculated. Pink and blue circles represented genes differentially expressed in HD018 and “Yudal,” respectively. Green part obtained by the superposition of pink and blue circles represents the differentially expressed genes common to both genotypes. In green part, only the genes with same type of regulation are considering. For each Venn diagram, the genotype with the higher amount of DEGs is represented by a circle of the same size (maximum size, independently of the number of gene). Then, the sizes of the two other color (other genotype and common part) are proportional to the biggest circle based on their amounts of genes included into. The numbers show exactly how many genes are upregulated or downregulated for each of the three colored parts.

When the plants have not been inoculated, N1 fertilization in HD018 resulted in the transcriptional regulation of 3,443 genes at 14 dpi and 4,246 genes at 27 dpi (Figure 4). Under the same condition, 3,537 genes at 14 dpi and 7,907 genes at 27 dpi were transcriptionally regulated in “Yudal.” Among the sets of DEGs, 34 and 27% were common to both genotypes, at 14 and 27 dpi, respectively.

Under inoculated condition, N1 fertilization in HD018 resulted in the transcriptional regulation of 3,217 genes at 14 dpi and 5,288 genes at 27 dpi (Figure 4). Under N1, 4,806 genes at 14 dpi and 5,870 genes at 27 dpi were transcriptionally regulated in “Yudal.” Among the sets of DEGs, 29 and 16% were common to both genotypes, at 14 and 27 dpi, respectively.

A singular enrichment analysis using AgriGo highlighted an enrichment of N1-upregulated genes for GO categories associated with ion transport and regulation of nitrogen metabolism. N1-downregulated genes were enriched for GO categories associated with a wider range of processes related to primary metabolism and ion transport. A detailed list of the genes associated with those GO terms and that were differentially expressed in our dataset are given in the Supplementary Table S3B.

A surprising low number of genes connected to nitrogen homeostasis were induced under N1, most significant being: *BnaC07g36810D* and *BnaA03g44820D* (encoding NRT1-8), *BnaC03g56990D* (encoding NRT1-5), and *BnaC02g06830D* (encoding an amino transporter homologue to *At5G16740*). N1 treatment also resulted in the expression of three cytosolic copper/zinc-SUPEROXIDE DISMUTASE 1 encoding genes (*BnaA09g48720D*, *BnaC08g42970D*, and *BnaC05g06430D*), and two cupredoxin encoding genes (*BnaA06g32330D* and *BnaC07g24070D*). Besides, a remarkably high number of genes unambiguously related to photosynthetic processes were induced under low-nitrogen condition in both genotypes. This included >100 genes encoding for a variety of photosystem I subunits, light harvesting complex proteins, small subunit 1 from RUBISCO, PSII oxygen evolving complex. However, even if the induction of these genes under N1 being statistically significant, their absolute transcription levels remained low (cpm < 6).

Among the most noticeable N1-repressed genes in the two genotypes were the four *B. napus* copies encoding FSD1 (a chloroplastic Fe-superoxide dismutase), two out of the four copies encoding the iron-metalloreductase FRO5, and the two copies encoding the nicotianamine-metal transporter YSL2. Two from the three *B. napus* genes encoding the glucosidase BGL1, involved in ABA remobilization were repressed. N1 also led to the repression of the two LOX2 encoding genes *BnaA07g38550D* and *BnaCnng38630D*, the JAR1 encoding gene *BnaA03g21360D* (involved in the synthesis of Jasmonoyl-isoleucine bioactive conjugate hormone), and two of the three 11/12-hydroxyjasmonate sulfotransferase 2A (ST2A) encoding genes, thus highlighting a possible regulation of some aspects of jasmonate signaling in long-term N1-deprived roots.

**Figure 5:**
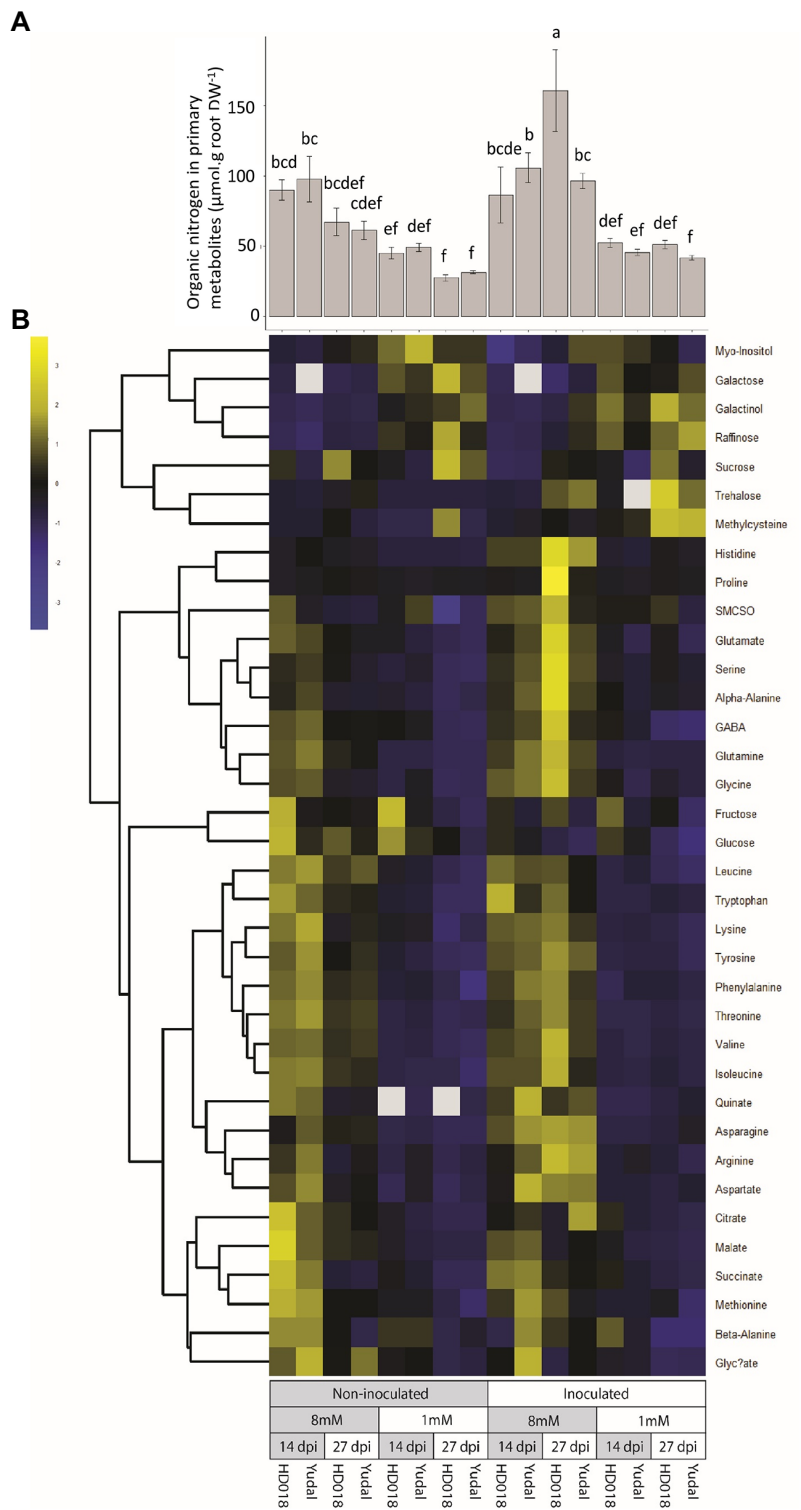
Primary metabolites (amino acids, non-structural carbohydrates, polyols, and organic acids) regulation in response to nitrogen supply and *Plasmodiophora brassicae* infection. **(A)** Organic nitrogen content in primary metabolites. Standard error are represented. Values followed by the same letter (a, b, c, d, e, f) are not significantly different (*p*<0.05) by Duncan’s multiple range test. **(B)** Primary metabolites content. Data of metabolites content were centered and reduced.

To further investigate the response of the two genotypes to variations in nitrogen supply, primary metabolite profiling was analyzed in root samples. From the concentration of these primary metabolites, we calculated the organic nitrogen concentration. Organic nitrogen in primary metabolite concentration was higher in the N8 condition compared to N1 condition (Figure 5A) both in the two genotypes and in the inoculated and non-inoculated plants. Moreover, no significant difference was detected between inoculated “Yudal” and HD018 under N1 at the two time-course points. In more details, very few metabolic differences were identified between the two non-inoculated genotypes (Figure 5B). In both “Yudal” and HD018, high-nitrogen supply was associated with elevated root concentrations of a series of 12 amino acids (β-alanine, asparagine, tryptophan, methionine, leucine, phenylalanine, glutamine, threonine, valine, isoleucine, lysine, tyrosine) and four other primary metabolites (glyceric acid, quinic acid, succinic acid, and ammonium). Low-nitrogen supply was associated with the accumulation of galactose, galactinol, raffinose, and myo-inositol. Thus, from a metabolic point of view, both genotypes displayed very similar responses to nitrogen deficiency in absence of pathogen infection.

Metabolic profiling also highlighted in both inoculated genotypes a clubroot-triggered accumulation of trehalose and S-methyl-cysteine, two marker metabolites of clubroot infection. Both metabolites were accumulated at a lower extent in “Yudal” under N1, compared to the three other inoculated modalities. However, we did not identify any pathogen-triggered modification of primary metabolite concentration related to low-nitrogen induced resistance response in “Yudal.”

### A Set of Genes Associated With Nitrate Assimilation Processes Was Specifically Induced in “Yudal” by the Dual Constraint Low-Nitrogen Supply × *P. brassicae* Inoculation

A set of DEGs associated with the resistance-induced in “Yudal” under low-nitrogen supply was selected according to three differential comparisons, two by two: “Yudal” N1 inoculated versus (i) “Yudal” N8 inoculated, (ii) HD018 N1 inoculated, and (iii) HD018 N8 inoculated. To be selected, genes had to show significant differential expression and a log2FC lower than −1 or higher than 1 and the same regulation pattern in the three comparisons.

At 14 dpi, 259 DEGs were selected: 88 were expressed at a higher level and 171 were expressed at lower level in “Yudal” N1 inoculated compared to the other inoculated samples. At 14 dpi, no biological process was enriched in the set of genes specifically induced by infection in “Yudal” N1. At this time point, genes specifically repressed by infection in “Yudal” N1 were enriched in functions associated with defense and responses to pathogens.

At 27 dpi, 945 DEGs were selected: 451 were expressed at higher level, and 494 at lower level in “Yudal” N1 inoculated compared to all other conditions. Among these genes, 24 were upregulated and 42 were downregulated at both time-course points in “Yudal” N1. The specific inhibition of defense-related function in “Yudal” N1 observed at 14 dpi was not any more observed at 27 dpi. At this time point, the set of 451 genes specifically induced in “Yudal” was enriched in functions related to nitrate response and nitrate transport. This included a series of genes homologous to the “classical series” of Arabidopsis nitrate-related genes: inducible high-affinity nitrate transporters NRT2.1, NRT2.2, and NRT2.3 (9 copies), an interaction partner of NRT2, NAR2.1 (5 copies), nitrate transporters NRT1.1 and NRT1.7 (2 copies), ammonium transporters AMT1 (2 copies), nitrate reductase encoding genes NR (2 copies), and nitrite reductases NIR (3 copies).

**Figure 6:**
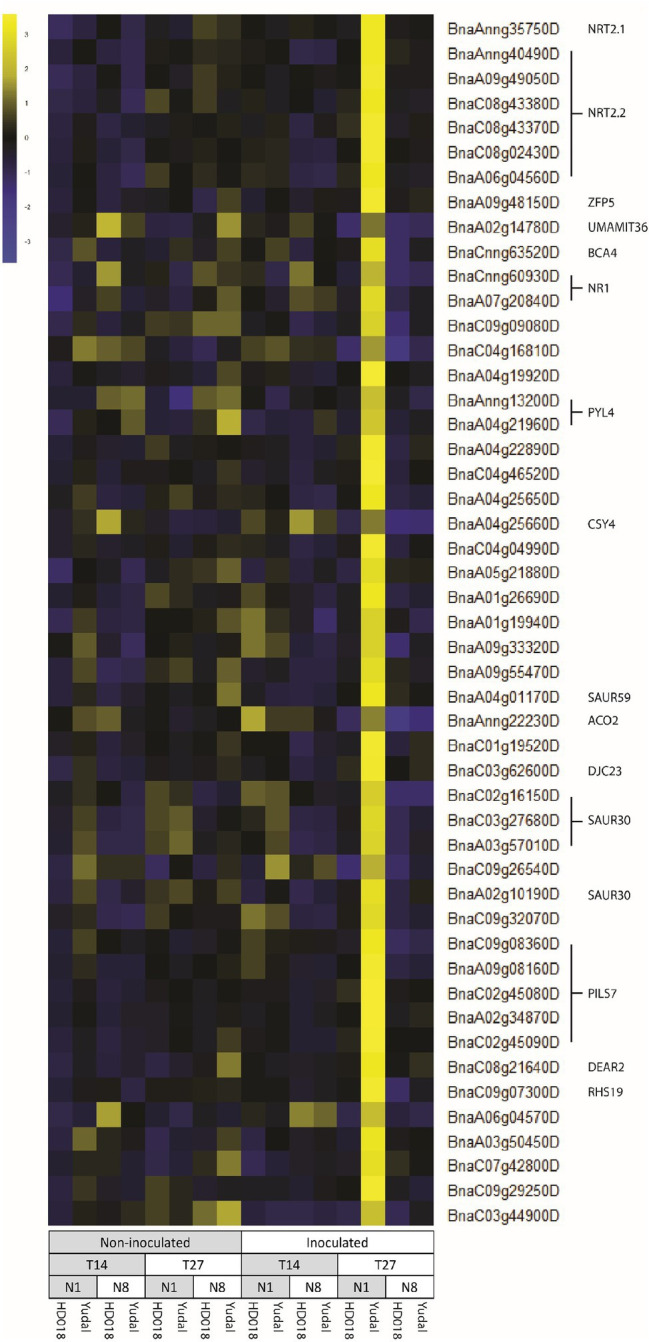
List of the 50 genes with the higher correlation value according to the regulation pattern of BnaAnng35750D. BnaAnng35750D was firstly identified for its huge induction in “Yudal” 1 mM inoculated compared to all the other samples. The genes expression data were centered and reduced. Gene function are based on *Arabidopsis thaliana* database, using gene homology with *B. napus*.

Among these 451 genes, the regulation pattern of NRT2 copies was atypical (Figure 6). These genes were specifically induced in “Yudal” N1 inoculated condition at 27 dpi compared to all the other conditions. A similar pattern of regulation was also detected for a set of genes, including genes related to auxin response, among which an auxin efflux carrier family protein, PILS7 (5 copies), and two genes encoding auxin response proteins, SAUR30 (1 copy) and SAUR59 (1 copy).

## Discussion

Based on the strong link between the pathogen nutritional requirements of an obligate parasite and the plant host metabolism, plant genetic variation influencing nitrogen use efficiency could theoretically influence the level of susceptibility/resistance of plants cultivated under low-nitrogen input (Ballini et al., 2013). In this perspective, experimental attempts to decipher the molecular basis of nitrogen-driven resistance may meet molecular processes involved in basic plant nitrogen assimilation. In non-inoculated plants under low-nitrogen condition we observed an important modulation of the primary metabolism. The reduction of many amino acids and few other primary metabolites (glyceric acid, quinic acid, succinic acid, and ammonium) content under low-nitrogen was previously described in non-inoculated *B. napus* leaves and roots (Gerendas and Sattelmacher, 1999; Tilsner et al., 2005). However, in our study, we observed only minor differences between the two *B. napus* genotypes, which cannot explain the large difference in term of symptoms level. The transcriptional response to nitrate, observed in the present work, was difficult to compare with other works since most of the available studies concern early responses (few minutes to hours after treatment) of *A. thaliana* to nitrate (reviewed in Canales et al., 2014). None out of the top 50 most consistent genes regulated in early response to nitrate highlighted by Canales et al. (2014), based on 11 *A. thaliana* studies, were observed in our study. By contrast, several genes identified as induced or repressed at a later stage after 10 days of nitrate deficiency (Krapp et al., 2011), were similarly regulated in our dataset. Most strikingly was the large repression of Fe-Superoxide Dismutase encoding genes (*At4g25100* in *Arabidopsis* and its four homologous in *B. napus*), and the induction of NRT2.5 encoding genes (*At1g12940* and *B. napus* homologous genes) and genes encoding a nodulin MtN21-EamA_like protein (*At1g21890* and *B. napus* homologous genes). The similarities between *Arabidopsis* and oilseed rape for long-term responses to nitrogen deficiency are particularly interesting for future functional studies. In addition to the global effect of nitrogen supply on the primary metabolite concentration, we observed two different patterns in non-inoculated and inoculated samples under high nitrogen. In fact, the global concentration of primary metabolites was highest in non-inoculated samples at 14 dpi, while it was highest in inoculated roots samples at 27 dpi. A higher concentration of primary metabolites, especially the amino acid, was previously observed in the genotype inoculated by *P. brassicae* at 42 dpi with the highest DI (Wagner et al., 2012). The increase of primary metabolites concentration in roots according to the susceptibility level was attributed to *P. brassicae* biotrophic life style. However, in our study, “Yudal” resistance cannot be explained by differences in both organic nitrogen and/or primary metabolite concentrations because they are similar between susceptible and resistant samples under low-nitrogen condition.

Plant hormones, such as jasmonate or salicylate, play a major role in plant defense mechanism against clubroot. Jasmonate responses have been previously identified in basal defense to clubroot in the *Arabidopsis* accession Col-0 (Gravot et al., 2012; Lemarié et al., 2015). The role of SA responses in partial resistance to clubroot have been previously reported in *Arabidopsis* accession Bur-0 (Jubault et al., 2013; Lemarié et al., 2015). Moreover, several works have reported that exogenous SA and methyl jasmonate (MeJA) treatment induces a significant reduction of clubroot symptoms in various *Brassica* (Agarwal et al., 2011; Lovelock et al., 2013; Lemarié et al., 2015; Xi et al., 2021). In the present study, *P. brassicae* inoculation led to an induction of SA-related markers and an accumulation of SA in both genotypes. This SA response was combined with the absence of JA response, as expected due to the antagonism between these two hormonal pathway. Nevertheless, no significant difference was observed in terms of SA accumulation between susceptible and resistant genotypes under low-nitrogen condition. According to the previous results and due to similar level of PR genes upregulation in the inoculated samples, SA response appears to be associated with the basal resistance in both “Yudal” and HD018. Although, we finally conclude that SA response level was not responsible of the low-nitrogen conditional resistance of “Yudal.” The present work however still offers a comprehensive view on the *B. napus* repertoire of SA-driven genes involved during clubroot infection.

Our work led to the identification of a set of genes, whose regulation pattern was linked to the resistance-triggered by low-nitrogen condition in “Yudal.” This set of genes comprised nitrate transporters (*NRT2.1*, *NRT2.2*, and *NRT3.1*), nitrate reductase (*NR1*), and auxin-related genes (*PILS7*, an auxin efflux carrier family protein, and *SAUR30* and *SAUR59*, two small auxin upregulated RNA). NRT2 proteins were firstly described for their implication in the nitrate influx in low N concentration condition. In *A. thaliana*, NRT2.1 appears to be by far the main component of the high-affinity transport systems (HATS) for nitrate root uptake under most conditions, with exception of severe N starvation (Cerezo et al., 2001; Filleur et al., 2001). NRT2 proteins are generally unable to transport nitrate on their own, but need to interact with the partner protein *NRT3.1* (Kotur et al., 2012; O’Brien et al., 2016). In addition to the nitrate transport function, nitrate transporters have been evidenced to be involved in nitrate sensing (Ho et al., 2009; Gojon et al., 2011). NRT2.1 has been shown to modify lateral root development independent of nitrate transport and thus act as a transceptor (Little et al., 2005). Two *NRT2* genes have been also identified as players in plant defense responses, *NRT2.1* and *NRT2.6*. A deletion in the high-affinity nitrate transporter *NRT2.1* in Arabidopsis resulted in a reduced susceptibility to *Pseudomonas syringae* by two different mechanisms, SA priming and interference with effector-triggered susceptibility (Camañes et al., 2012). In Arabidopsis also, *NRT2.6* was found induced by the phytopathogenic bacterium *Erwinia amylovora* (Dechorgnat et al., 2012). Interestingly, plants with a decreased *NRT2.6* expression showed a lower tolerance to pathogen attack. A correlation was found between *NRT2.6* expression and ROS species accumulation in response to infection by *E. amylovora* and treatment with the redox-active herbicide methyl viologen, suggesting a probable link between NRT2.6 activity and the production of ROS in response to biotic and abiotic stress. No study has yet highlighted the roles of NRT2 genes in clubroot resistance.

Auxin, one of the most investigated classes of plant hormones, is involved in many aspects of plant growth and development. Many studies suggest a pivotal role of auxin in primary root, lateral root, and root hair development (Osmont et al., 2007; Benfey et al., 2010; Overvoorde et al., 2010). Auxin transporters, as PILS7, were implicated in gradient formation or local accumulation to induced auxin response. Moreover, the auxin response during clubroot infection was highlighted by transcriptomics (Siemens et al., 2006; Jubault et al., 2013; Irani et al., 2018; Ciaghi et al., 2019; Robin et al., 2020) and metabolomics studies (Raa, 1971; Butcher et al., 1974; Kavanagh and Williams, 1981; Rausch et al., 1983; Ludwig-Müller et al., 1996; Grsic et al., 1999; Ugajin et al., 2003). Besides, the role of auxin has been constantly reported as a major feature of clubroot disease, but only a paucity of works has reported that allelic variations affecting auxin signaling could influence clubroot development. Thus, these genes are interesting candidates for future functional studies.

Previous genetic linkage analysis of clubroot resistance in a “Darmor-*bzh*” × “Yudal” double haploid progeny, identified resistance QTL against eH isolate of *P. brassicae* based on disease index (Laperche et al., 2017). The QTL C02 may play an important role in the nitrogen-drive modulation of “Yudal” resistance compared to HD018. Indeed, among all the QTL that were identified as influencing resistance to eH isolate in the “Darmor-*bzh*” × “Yudal” progeny, this QTL C02 was the only one for which there is an allelic variation between “Yudal” and the recombinant line HD018 (Laperche et al., 2017; Aigu et al., 2018). However, none of the 341 genes included in the C02 QTL confidence interval was common with the list of DEGs associated with the resistance-induced in “Yudal” under low-nitrogen supply (291 genes at 14 dpi and 945 genes at 27 dpi). Moreover, none of these 341 genes had any regulation pattern that can be related to “Yudal” lower symptoms (no genes specifically induced/repressed under low-nitrogen supply in Yudal whatever the inoculation condition). Further analyses would be required to identify every sequences variations between the two genotypes in the C02 QTL interval and then explore if those variations could influence directly or indirectly the transcription of the selected candidate DEGs. Alternatively, these DEGs could be regulated by genetic variations at other QTL that may have been undetected in the genetic analyses from Laperche et al. (2017).

## Conclusion

To summarize, (1) Our data suggested that low-nitrogen conditional resistance in “Yudal” relied at first on less symptom (root galls) development. (2) Low-nitrogen supply exerted a strong but similar effect on amino acid and soluble carbohydrate concentrations in both genotypes. However, this effect was relatively weak on root transcriptional responses on the studied time-course points, compared to the impact of genotype and inoculation factors. About half of nitrogen-driven transcriptional regulations were common to “Yudal” and HD018, thus suggesting that the influence of nitrogen was similar in both plant genotypes. (3) Nitrogen constraint did not seem to exert *per se* any evident influence on constitutive defenses. (4) Low-nitrogen conditional resistance in “Yudal” was found associated with the induction of series of *NRT2.2* and genes related to inorganic nitrogen acquisition and to auxin-related genes. Additional work would be needed to further understand how these regulations inhibit the development of galls.

## Data Availability Statement

The original contributions presented in the study are publicly available. This data can be found at: Raw reads are available at the European Nucleotide Archive database system under the project accession number PRJEB44381 (samples ERS6263817 to ERS6263880).

## Author Contributions

MM-D, AG, YA, SD, and AL designed the experiments. Metabolic profiling was performed by NM and YA. YA, SD, CL, KG, and AG contributed to clubroot assays and sampling. Sample processing and RNA extraction were performed by KG, CL, NM, and YA. RNA-seq analysis and bioinformatics were performed by YA and FL. YA, AG, and MM-D wrote the manuscript, with advices from AL, KG, and SD. All authors read and approved the final manuscript.

## Funding

This study was funded by the French Association for the Promotion of Oilseed Crops Breeding (PROMOSOL). The doctoral followship of YA was funded by Université de Rennes 1.

## Conflict of Interest

The authors declare that the research was conducted in the absence of any commercial or financial relationships that could be construed as a potential conflict of interest.

## Publisher’s Note

All claims expressed in this article are solely those of the authors and do not necessarily represent those of their affiliated organizations, or those of the publisher, the editors and the reviewers. Any product that may be evaluated in this article, or claim that may be made by its manufacturer, is not guaranteed or endorsed by the publisher.

## Supplementary Material

Supplementary Table S1 Number of paired-end reads sequenced for each sample.

Supplementary Table S2 Summary of differentially expressed gene number according to the three main studied factors: genotype, inoculation, and nitrate supply.

Supplementary Table S3 List of differentially expressed genes according to inoculation (S3A), and nitrate supply (S3B).

Supplementary Figure S1 Distribution of gene expression in the 64 samples.

Supplementary Figure S2 MultiDimensional Scaling (MDS) plot of RNA-seq data.

Supplementary Figure S3 Absence of jasmonate (JA) response induced by *Plamodiophora brassicae* inoculation.
