# Supplementary material for: Multi-Omic Investigation of Low-Nitrogen Conditional Resistance to Clubroot Reveals Brassica napus Genes Involved in Nitrate Assimilation
Source: Front Plant Sci. 2022 Feb 11;13:790563. doi: 10.3389/fpls.2022.790563 (PMC8874135; doi:10.3389/fpls.2022.790563)
Supplement: Supplementary Figure S1 — Distribution of gene expression in the 64 samples. [file Presentation_1.PPTX]

## Slide 1
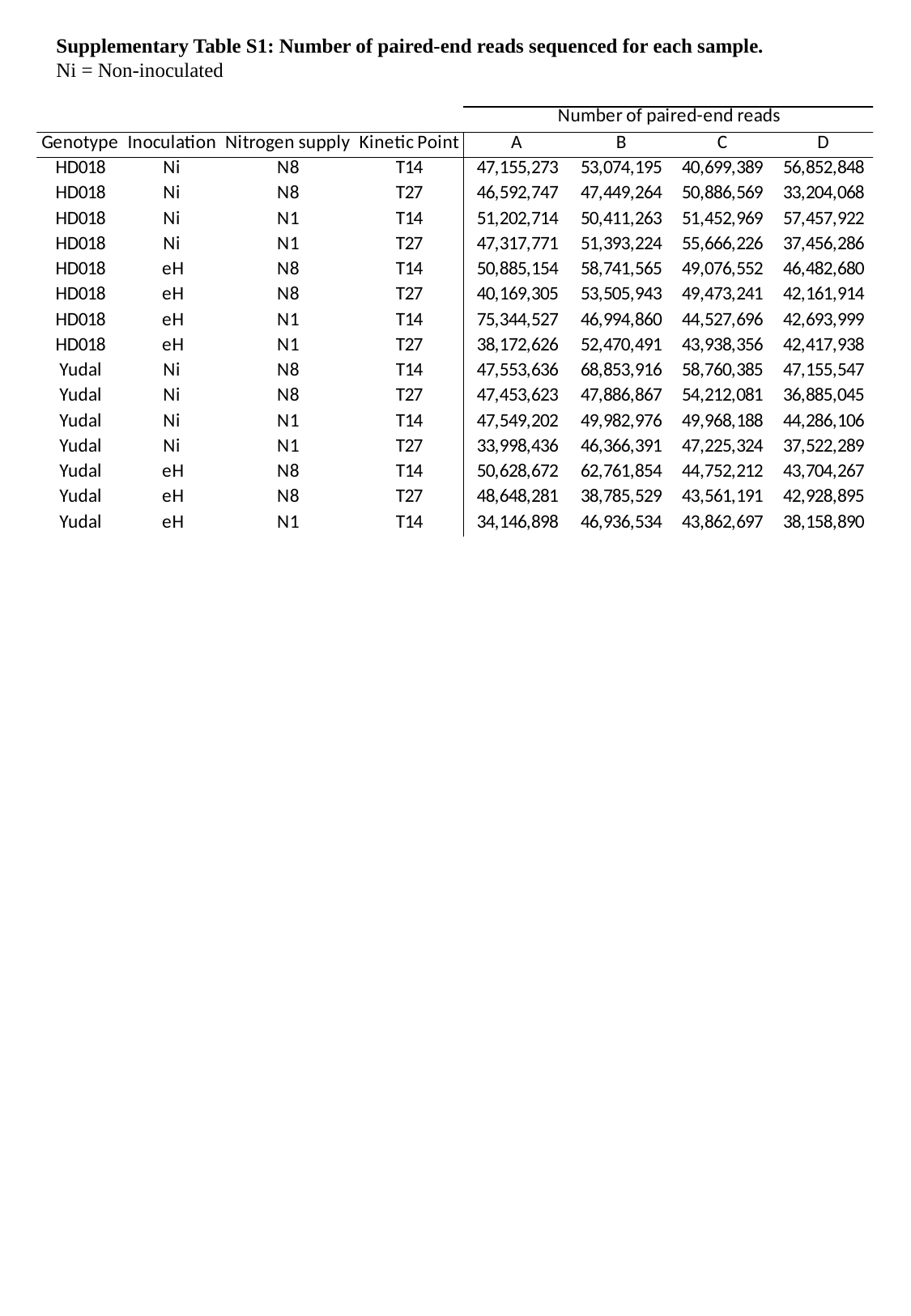

Supplementary Table S1: Number of paired-end reads sequenced for each sample.
Ni = Non-inoculated

## Slide 2
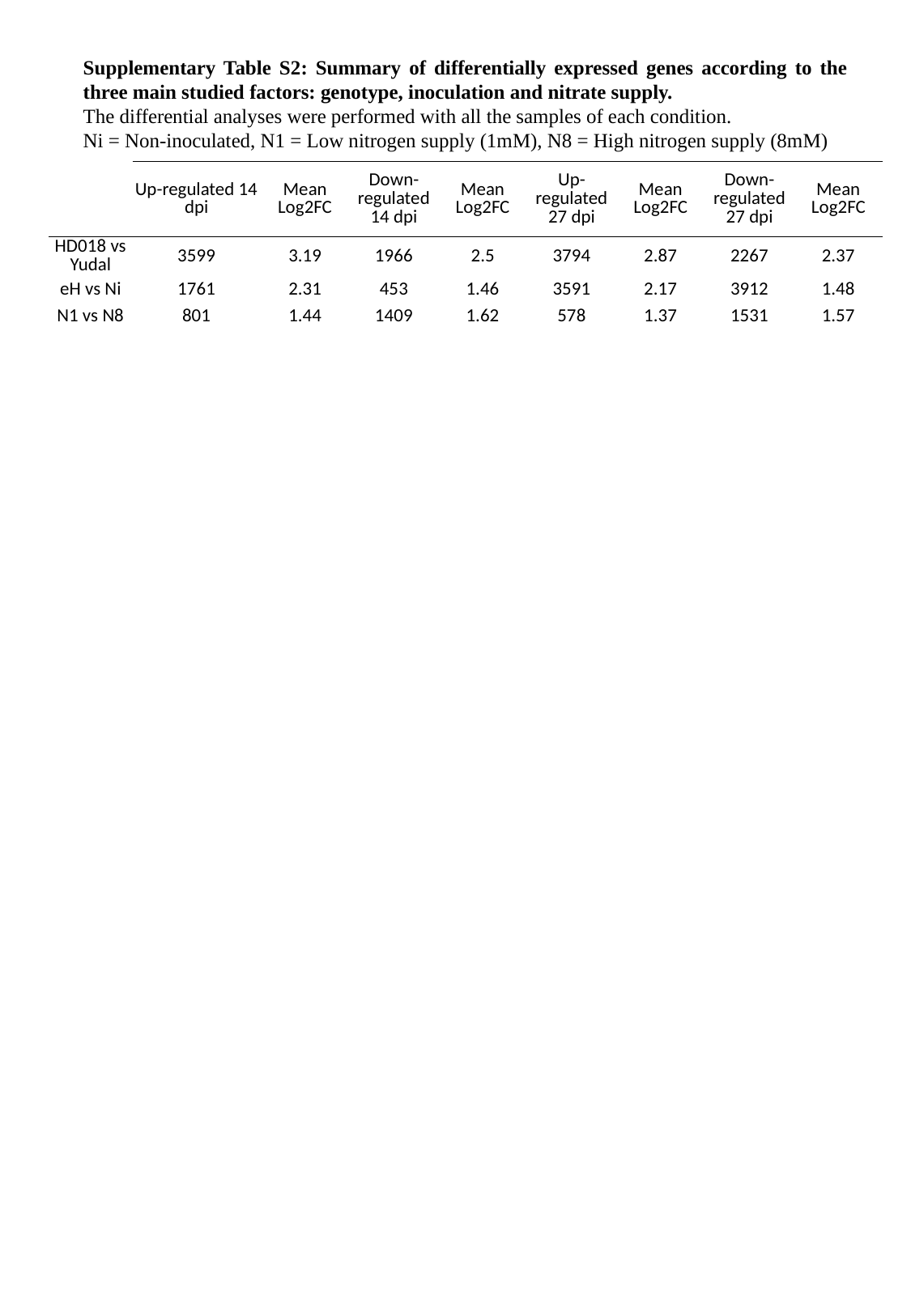

Supplementary Table S2: Summary of differentially expressed genes according to the three main studied factors: genotype, inoculation and nitrate supply.
The differential analyses were performed with all the samples of each condition.
Ni = Non-inoculated, N1 = Low nitrogen supply (1mM), N8 = High nitrogen supply (8mM)
| | Up-regulated 14 dpi | Mean Log2FC | Down-regulated 14 dpi | Mean Log2FC | Up-regulated 27 dpi | Mean Log2FC | Down-regulated 27 dpi | Mean Log2FC |
| --- | --- | --- | --- | --- | --- | --- | --- | --- |
| HD018 vs Yudal | 3599 | 3.19 | 1966 | 2.5 | 3794 | 2.87 | 2267 | 2.37 |
| eH vs Ni | 1761 | 2.31 | 453 | 1.46 | 3591 | 2.17 | 3912 | 1.48 |
| N1 vs N8 | 801 | 1.44 | 1409 | 1.62 | 578 | 1.37 | 1531 | 1.57 |

## Slide 3
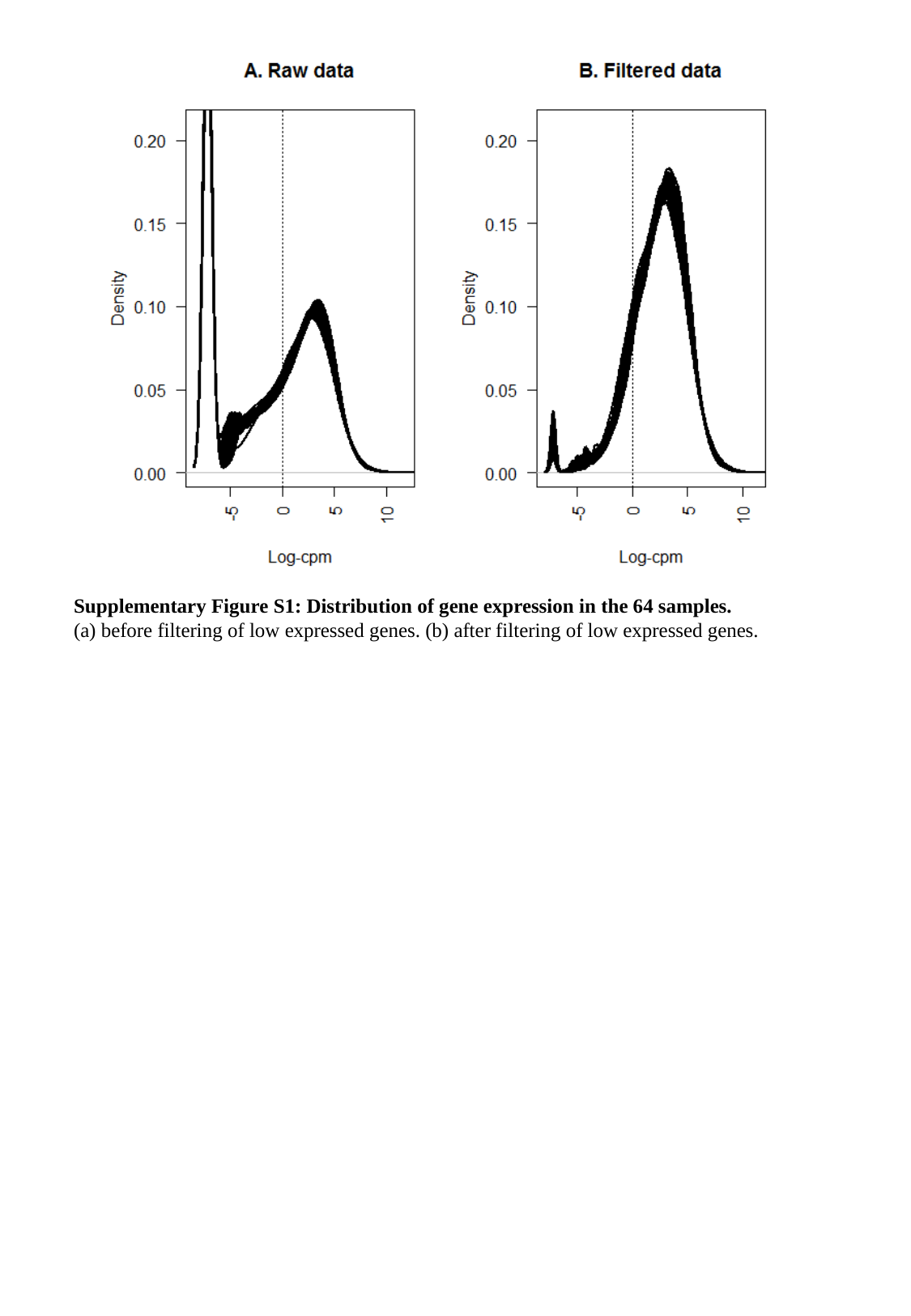

Supplementary Figure S1: Distribution of gene expression in the 64 samples.
(a) before filtering of low expressed genes. (b) after filtering of low expressed genes.

## Slide 4
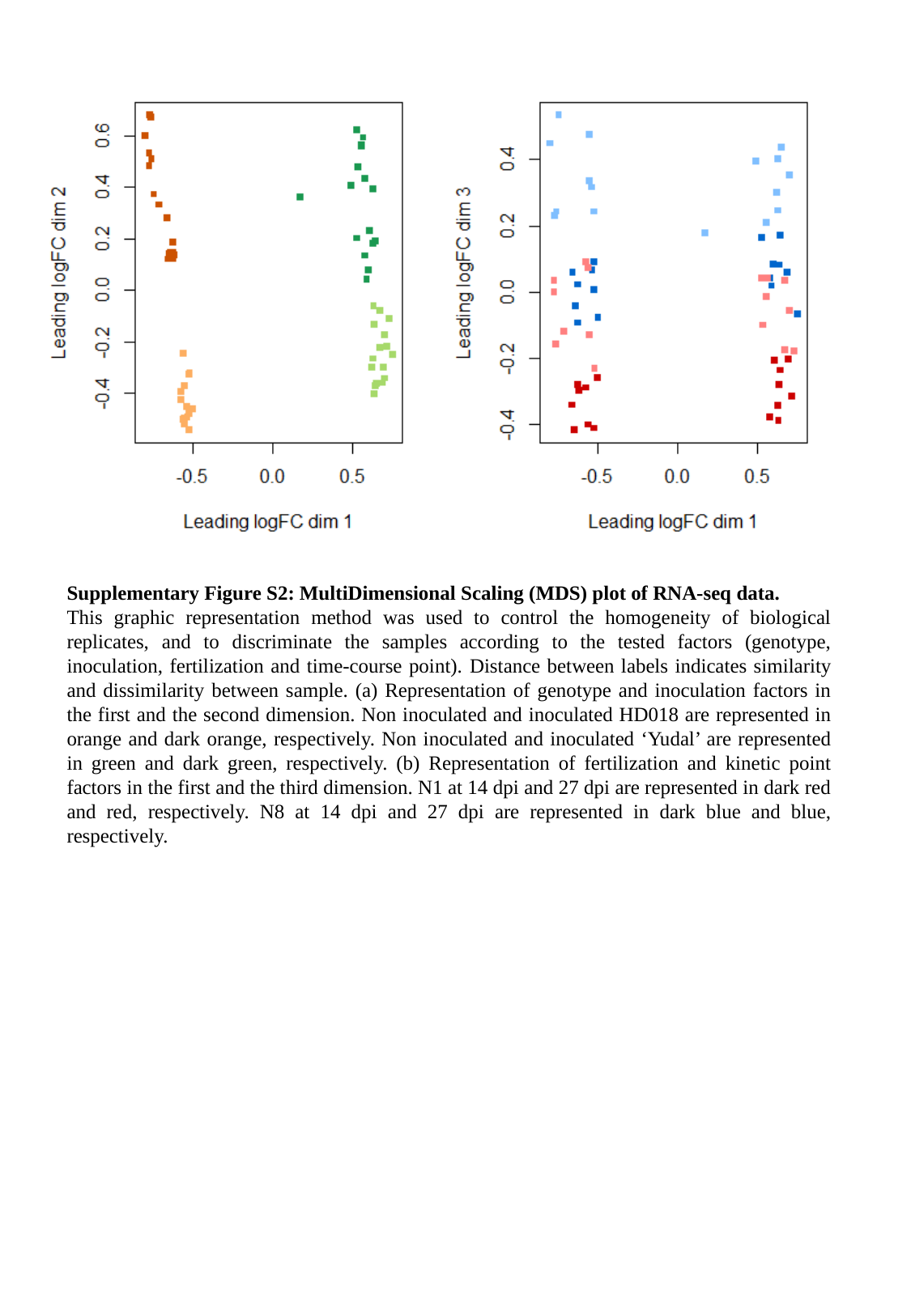

Supplementary Figure S2: MultiDimensional Scaling (MDS) plot of RNA-seq data.
This graphic representation method was used to control the homogeneity of biological replicates, and to discriminate the samples according to the tested factors (genotype, inoculation, fertilization and time-course point). Distance between labels indicates similarity and dissimilarity between sample. (a) Representation of genotype and inoculation factors in the first and the second dimension. Non inoculated and inoculated HD018 are represented in orange and dark orange, respectively. Non inoculated and inoculated ‘Yudal’ are represented in green and dark green, respectively. (b) Representation of fertilization and kinetic point factors in the first and the third dimension. N1 at 14 dpi and 27 dpi are represented in dark red and red, respectively. N8 at 14 dpi and 27 dpi are represented in dark blue and blue, respectively.

## Slide 5
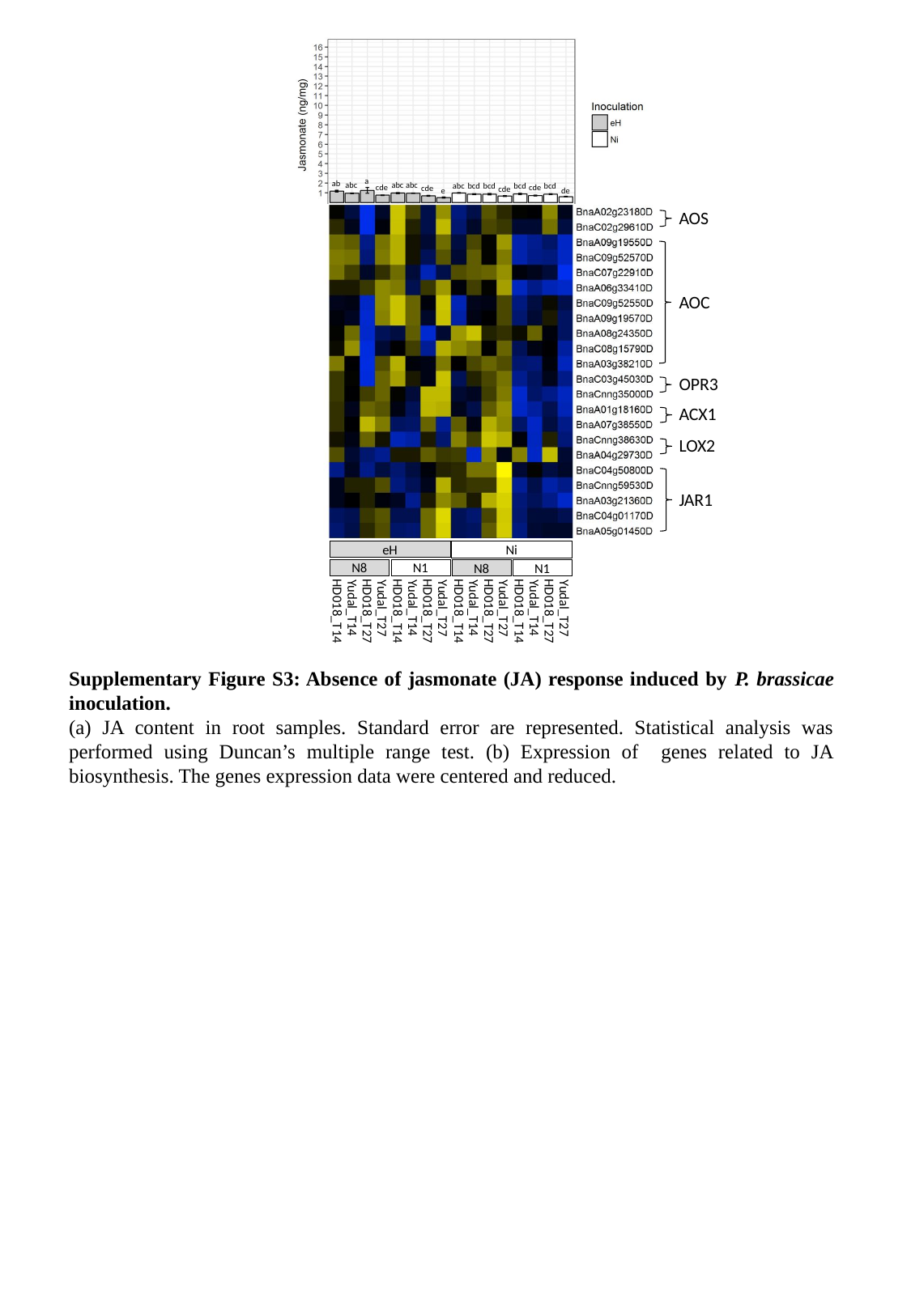

a
ab
abc
abc
abc
bcd
bcd
bcd
abc
bcd
cde
cde
cde
cde
de
e
AOS
AOC
OPR3
ACX1
LOX2
JAR1
eH
Ni
N8
N1
N8
N1
HD018_T14
HD018_T27
HD018_T14
HD018_T27
HD018_T14
HD018_T27
HD018_T14
HD018_T27
Yudal_T14
Yudal_T14
Yudal_T14
Yudal_T14
Yudal_T27
Yudal_T27
Yudal_T27
Yudal_T27
Supplementary Figure S3: Absence of jasmonate (JA) response induced by P. brassicae inoculation.
(a) JA content in root samples. Standard error are represented. Statistical analysis was performed using Duncan’s multiple range test. (b) Expression of genes related to JA biosynthesis. The genes expression data were centered and reduced.
